# Health professionals’ practices related with tourniquet use during peripheral venipuncture: a scoping review *

**DOI:** 10.1590/1518-8345.2743-3125

**Published:** 2019-04-29

**Authors:** Anabela de Sousa Salgueiro-Oliveira, Paulo Jorge dos Santos Costa, Luciene Muniz Braga, João Manuel Garcia Nascimento Graveto, Vânia Silva Oliveira, Pedro Miguel Santos Dinis Parreira

**Affiliations:** 1 Escola Superior de Enfermagem de Coimbra , Unidade de Investigação em Ciências da Saúde: Enfermagem , Coimbra , Portugal .; 2 Universidade Federal de Viçosa , Departamento de Medicina e Enfermagem , Viçosa , MG , Brasil .

**Keywords:** Tourniquets, Catheterization, Peripheral, Equipment Contamination, Cross Infection, Health Personnel, Decontamination, Torniquetes, Cateterismo Periférico, Contaminação de Equipamentos, Infecção Hospitalar, Pessoal de Saúde, Descontaminação, Torniquetes, Cateterismo Periférico, Contaminación de Equipos, Infección Hospitalaria, Personal de Salud, Descontaminación

## Abstract

**Objectives:**

during peripheral venipuncture, health professionals are recommended to use a tourniquet above the puncture site in order to potentiate venous distension. Given its characteristics and use in clinical settings, tourniquets may represent a source of microorganism dissemination. However, the results of scientific studies in this area are scattered in the literature. This scoping review aims to map the available evidence on health professionals’ practices related with tourniquet use during peripheral venipuncture and associated microbiological contamination.

**Methods:**

scoping review following the Joanna Briggs Institute methodology. Two independent reviewers analyzed the relevance of the studies, extracted and synthesized data.

**Results:**

fifteen studies were included in the review. Overall, tourniquets were reused without being subject to recurring decontamination processes. It has been found that practitioners share these devices among themselves and use them successively for periods between two weeks and seven and half years.

**Conclusion:**

nursing practices related to tourniquet use during peripheral venipuncture are not standard. Reuse of tourniquets may jeopardize the patient’s safety if reprocessing (cleaning and disinfection/sterilization) is not adequate, given the type of tourniquet material and microbiota found. New studies are needed to assess the impact of various types of reprocessing practices on tourniquet decontamination and patient safety.

## Abstract (pt)

**Objetivos:**

durante a punção venosa periférica, recomenda-se o uso de um garrote acima do local da punção para potencializar a distensão venosa. Dadas as suas características e o uso em ambientes clínicos, os garrotes podem representar uma fonte de disseminação de micro-organismos. Entretanto, os resultados de estudos científicos nessa área estão dispersos na literatura. Esta revisão de escopo tem como objetivo mapear as evidências disponíveis a respeito das práticas dos profissionais de saúde no que concerne ao uso do garrote durante a punção venosa periférica e à contaminação microbiológica associada.

**Método:**

revisão de escopo de acordo com a metodologia do Instituto Joanna Briggs. Dois revisores independentes analisaram a relevância dos estudos, extraíram e sintetizaram dados.

**Resultados:**

quinze estudos foram incluídos na revisão. Em geral, os garrotes foram reutilizados sem processos de descontaminação recorrentes. Verificou-se que os profissionais compartilham esses dispositivos entre si e os usaram continuamente por períodos entre duas semanas e sete anos e meio.

**Conclusão:**

as práticas de enfermagem relacionadas ao uso do garrote durante a punção venosa periférica não são uniformes. A reutilização de garrotes pode colocar em risco a segurança do paciente se o reprocessamento (limpeza e desinfecção/esterilização) não for adequado, dado o tipo de material do garrote e a microbiota encontrada. Novos estudos são necessários para avaliar o impacto de vários tipos de práticas de reprocessamento na descontaminação de garrotes e na segurança do paciente.

## Abstract (es)

**Objetivos:**

durante la punción venosa periférica, se recomienda el uso de un garrote arriba del sitio de la punción para potenciar la distensión venosa. Dadas sus características y uso en ambientes clínicos, los garrotes pueden representar una fuente de diseminación de microorganismos. Sin embargo, los resultados de estudios científicos en esta área están dispersos en la literatura. Esta revisión de alcance tiene como objetivo mapear las evidencias disponibles acerca de las prácticas de los profesionales de salud en lo que concierne al uso del garrote durante la punción venosa periférica y la contaminación microbiológica asociada.

**Método:**

revisión de alcance de acuerdo con la metodología del Instituto Joanna Briggs. Dos revisores independientes analizaron la relevancia de los estudios, extrajeron y sintetizaron datos.

**Resultados:**

quince estudios se incluyeron en la revisión. En general, los garrotes fueron reutilizados sin procesos de descontaminación recurrentes. Se verificó que los profesionales comparten estos dispositivos entre sí y los utilizaron continuamente por períodos entre dos semanas y siete años y medio.

**Conclusión:**

las prácticas de enfermería relacionadas al uso del garrote durante la punción venosa periférica no son uniformes. La reutilización de garrotes puede poner en riesgo la seguridad del paciente si el reprocesamiento (limpieza y desinfección/esterilización) no es adecuado, dado el tipo de material del garrote y la microbiota encontrada. Nuevos estudios son necesarios para evaluar el impacto de varios tipos de prácticas de reprocesamiento en la descontaminación de garrotes y en la seguridad del paciente.

## Introduction

Peripheral venipuncture, for the catheterization of a vascular access or blood collection, constitutes one of the most frequent and invasive clinical procedures perform in healthcare settings ^(^ ^1^ ^-^ ^3^ ^)^ . In order to stop blood flow and promote vascular distension, the use of a tourniquet for a period not exceeding 60 seconds is recommended ^(^ ^4^ ^)^ . For this purpose, these medical devices should be applied at a distance of 5 to 10 centimeters above the desired puncture site ^(^ ^5^ ^)^ .

Medical devices contamination is a major public health concern, since their extensive reprocessing and reuse between patients may hamper the care provided ^(^ ^6^ ^)^ . Several studies show that highly portable medical devices, such as tourniquets, are associated with high contamination rates, often linked with bacterial cultures that are multidrug resistant to conventional antibiotic therapy ^(^ ^7^ ^-^ ^9^ ^)^ . However, there is evidence pointing to a wide gap between health professionals knowledge and practices in this field ^(^ ^10^ ^)^ .

Consequently, tourniquets used during peripheral venous puncture may be the source of dissemination of microorganisms, due to the irregular use of these specific medical devices, without complying with specific guidelines ^(^ ^11^ ^-^ ^12^ ^)^ . For this purpose, it is recommended that its manufacturing material presents a low risk of microbial contamination ^(^ ^11^ ^,^ ^13^ ^)^ . To break this chain of microorganism dissemination, most recent guidelines recommend the use of single-patient tourniquets ^(^ ^4^ ^)^ .

After an extensive review of the literature, no studies were found that synthesize the potential contamination of tourniquets used by professionals in procedures involving peripheral venipuncture, identifying inherent practices in their use. Additionally, the authors of the studies found that focused solely on the microbiological contamination of tourniquets identified as a limitation of their work the non-identification of inherent health professionals’ practices in their use ^(^ ^14^ ^-^ ^15^ ^)^ .

Given this scenario, a scoping review was conducted, guided by the methodology proposed by the Joanna Briggs Institute for Scoping Reviews ^(^ ^16^ ^)^ . This review intends to answer the following question: What are the current practices of health professionals related with tourniquet use during peripheral venipuncture, and associated microbiological contamination?

## Methods

The synthesis of evidence in systematic reviews is at the center of evidence-based practice ^(^ ^14^ ^)^ . Different objectives and review questions require the development of new approaches, such as scoping reviews, to synthesize evidence in a more effective and rigorous way ^(^ ^17^ ^)^ . The scoping review approach was selected because this type of review aims to map the existing evidence underpinning a research area and identify gaps in the existing evidence. It is a preliminary exercise that justifies and informs the development of a systematic literature review ^(^ ^16^ ^)^ . This methodology does not aim to analyze the methodological quality of included studies or find the best scientific evidence, but rather map the existing scientific evidence ^(^ ^16^ ^)^ .

Using the Participants, Concept, and Context (PCC) strategy, this scoping review included studies that focused on: a) as participants, health professionals with certified competences to perform peripheral venipuncture; b) as the concept, studies focusing on health professional’s practices regarding tourniquet use during peripheral venipuncture, with the additional analysis of microbiological contamination; c) all clinical and geographic settings as the context.

The search strategy included published and unpublished studies and was composed of three steps: i) Limited initial search in MEDLINE (via PubMed) and CINAHL complete (via EBSCO), followed by an analysis of text words in titles and abstracts and index terms used to describe the article; ii) Second search using all keywords and index terms identified in the included databases; iii) The references of all articles and reports found in the search were analyzed to identify additional studies. Studies written in English, Spanish, French and Portuguese were considered for inclusion in this review, regardless of the year of publication.

This review included as databases and repositories CINAHL Complete (via EBSCO), Cochrane Central Register of Controlled Trials, Scopus, OpenGrey, Scientific Electronic Library Online, *Repositórios Científicos de Acesso Aberto de Portugal, Portal de Periódicos da Coordenação de Aperfeiçoamento de Pessoal de Nível Superior (Capes)* , and the Joanna Briggs Institute Clinical Online Network of Evidence for Care and Therapeutics. Boolean logic was used with search terms including: (tourniquet OR tourniquets) AND (contamination OR colonization OR colonisation OR organism OR organisms OR infect* OR bacter* OR fung* OR virus OR viral OR viruses OR pathogenic OR pathogens OR yeast OR yeasts OR microorganism OR microorganisms OR spores OR “colony count” OR colonies OR colony OR “colony forming units” OR “colony forming unit” OR microbial OR fomite OR fomites OR cross-contamination OR cross-infection OR “Equipment Contamination”).

The relevance of the articles included in the review was analyzed by two independent reviewers based on the information provided in the title and abstract. Whenever the reviewers had doubts about the relevance of a study based on its abstract, the full-text version was obtained. Two reviewers independently examined the full-text version of the articles to check if they met the inclusion criteria. Disagreements between reviewers were resolved through discussion, or by a third reviewer.

The relevance of studies identified in reference lists was assessed based on their title and abstract. Two independent reviewers extracted the data using an instrument designed by the researchers, in line with the objective and question of the review. Disagreements between reviewers were resolved through discussion or by a third reviewer. Whenever necessary, the authors of primary studies were contacted with a view to obtaining more information and/or clarifying data.

## Results

**Figure 1:**
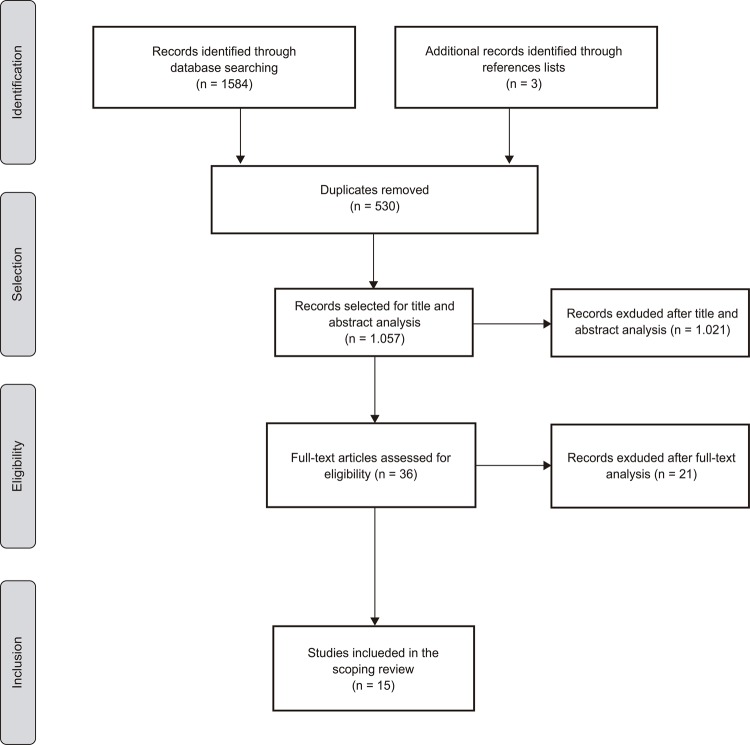
PRISMA Flow diagram (adapted) of the study selection process. Coimbra, Portugal, 2017

As shown in Figure 1, the search identified 1.587 potentially relevant studies. Of these, 530 were excluded for being duplicates. The remaining 1.057 articles were screened by title and abstract. Of these, 36 articles were included for full-text analysis by two independent reviewers.

**Figure 2:**
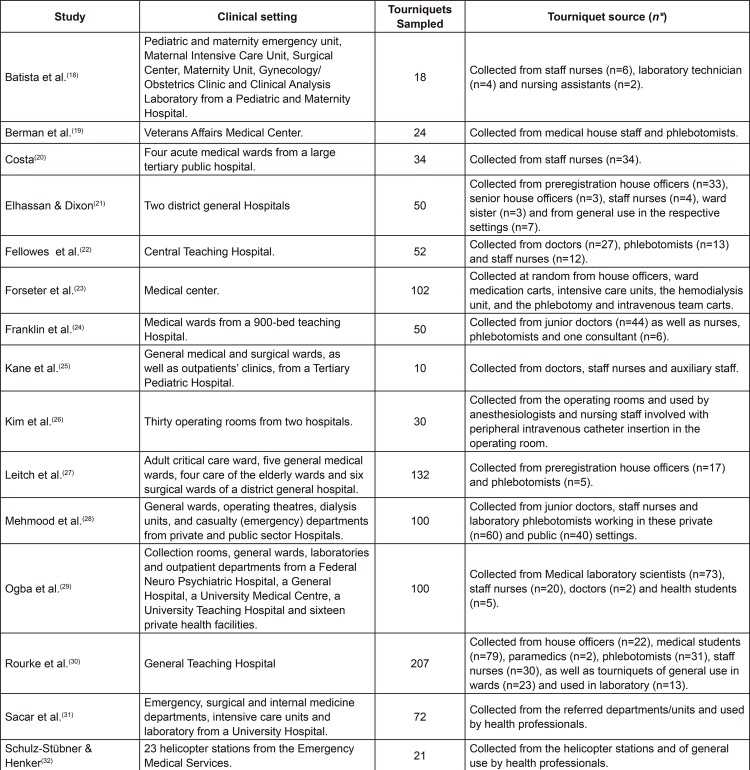
Clinical setting and tourniquet source. Coimbra, Portugal, 2017

Fifteen studies were excluded due to absence of data on health professional’s practices regarding tourniquet use during peripheral venipuncture, while six were excluded due to lack of full-text access and author’s reply. Therefore, 15 primary studies were included for data extraction and synthesis (Figure 2).

*Population Size

Of the included studies, six were conducted in the United Kingdom ^(^ ^21^ ^-^ ^22^ ^,^ ^24^ ^-^ ^25^ ^,^ ^27^ ^,^ ^30^ ^)^ and two in the United States of America ^(^ ^19^ ^,^ ^23^ ^)^ . With only one study per country, this scoping also includes studies carried out in Brazil ^(^ ^18^ ^)^ , Portugal ^(^ ^20^ ^)^ , South Korea ^(^ ^26^ ^)^ , Pakistan ^(^ ^28^ ^)^ , Nigeria ^(^ ^29^ ^)^ , Turkey ^(^ ^31^ ^)^ and Germany ^(^ ^32^ ^)^ . Studies were published between 1986 ^(^ ^17^ ^)^ and 2017 ^(^ ^18^ ^)^ .

All included studies collected diverse information regarding health professionals’ practices associated with tourniquet use during peripheral venipuncture. The most common method to collect this information was true questionnaire ^(^ ^19^ ^-^ ^24^ ^,^ ^26^ ^-^ ^32^ ^)^ , followed by observation ^(^ ^25^ ^,^ ^27^ ^,^ ^31^ ^)^ and structured interview ^(^ ^18^ ^)^ .

Overall, 1.104 tourniquets were collected, varying from 10 ^(^ ^25^ ^)^ to 241 ^(^ ^30^ ^)^ samples per study. Tourniquets were often owned by healthcare professionals such as doctors, staff nurses, phlebotomists and laboratory workers from all ranks and clinical specialties, or of general ward/laboratory use.

Regarding tourniquet characteristics, one study ^(^ ^21^ ^)^ show that 77% of the included participants used reusable tourniquets. With similar results, one study ^(^ ^20^ ^)^ identified that all the included tourniquets (100%) were reusable and made of fabric. As pointed out by other authors ^(^ ^18^ ^)^ , disposable gloves were used as tourniquets during peripheral venipuncture in neonates and infants. Moreover, in another study ^(^ ^29^ ^)^ all tourniquets were reusable, with 3% being made of elastic while the remaining were made of rubber. Furthermore, 97% of the health professionals reused their tourniquet and justified this due to the insufficient resources in their units ^(^ ^29^ ^)^ .

In one particular study ^(^ ^25^ ^)^ results show that 52% of the health professionals used elastic tourniquets, 39% used “human tourniquets” (application of manual pressure in the limb with or without assistant), 8% used plastic and wipeable tourniquets and 1% used gauze. However, after the introduction of single-use disposable tourniquets in the clinical settings, 52% carried using elastic tourniquets, 27% started using single-use disposable tourniquets and 20% continued applying manual pressure ^(^ ^25^ ^)^ .

Other authors ^(^ ^23^ ^)^ found that 96 of the 102 sampled tourniquets were made of latex and six were of rubber with Velcro fastener closures. Interestingly, one study ^(^ ^19^ ^)^ verified that medical house staff and phlebotomists, aside from Velcro-covered tourniquets, also commonly used Penrose drains as tourniquets.

Concerning time and frequency of use, five studies considered for how long tourniquets would be in the health professionals’ possession ^(^ ^19^ ^,^ ^21^ ^-^ ^22^ ^,^ ^24^ ^,^ ^30^ ^)^ . The lowest length of time found was two consecutive weeks, although some health professionals in the same study used their tourniquets for 104 consecutively weeks ^(^ ^22^ ^)^ . In the same study, the mean age for doctors’ tourniquets was 11 weeks, for nurses 93 weeks and for phlebotomists 32 weeks ^(^ ^22^ ^)^ . The lowest length of time was proximate with other included study, where health professionals used the same tourniquet continuously between three and 24 weeks ^(^ ^19^ ^)^ .

In another study ^(^ ^21^ ^)^ , the mean tourniquet age was 14 weeks, although one tourniquet was found to be used consecutively for one and half year. Moreover, other authors ^(^ ^24^ ^)^ found tourniquets that were being used continuously between two to 208 weeks (median of 39 weeks). Lastly, one particular study ^(^ ^30^ ^)^ found that the mean length of time tourniquets had been in a person’s possession was 1.86 years, with a range of three days to 7.5 years.

Five studies analyzed the number of times health professionals used these tourniquets during peripheral venipuncture on an average day ^(^ ^19^ ^,^ ^21^ ^-^ ^22^ ^,^ ^29^ ^)^ . In one study ^(^ ^19^ ^)^ , tourniquets were used with 15 to 20 patients per day, while in another research effort ^(^ ^21^ ^)^ this number decreased to three patients. With a similar range, authors in one study ^(^ ^22^ ^)^ found that health professionals used their tourniquets 11 patients per day, on average, ranging from one to 30 patients. This number was also verified in another study ^(^ ^29^ ^)^ , with 65% of health professionals using their tourniquet more than 20 patients per day, 15% between 16 to 20 patients, 11% between 11 to 15 patients, while 9% with 10 or less patients per day.

Although tourniquets used during peripheral venipuncture had been used consistently and repeatedly in clinical settings, health professionals only select new tourniquets if the previous one is lost ^(^ ^21^ ^,^ ^26^ ^,^ ^30^ ^)^ . However, authors in one study ^(^ ^30^ ^)^ found that 16% of the included participants replace their tourniquets if visible soiled. Nonetheless, 3% of the participants stated they didn’t replace this device voluntarily under any circumstance ^(^ ^30^ ^)^ .

In another study ^(^ ^20^ ^)^ , 82% of the participants stated they discarded reusable tourniquets if they were soiled with organic matter, 60% if they used them with infected patients and 50% after a period of usage individually perceived as excessive. However, 6% of professionals never contemplated discarding their tourniquets ^(^ ^20^ ^)^ .

Four studies analyzed the sharing of tourniquets with other professionals. In one study ^(^ ^20^ ^)^ 90% of participating nurses share their tourniquets with other nurses, 52% with doctors and 48% with other allied health professionals. Correspondingly, other authors found that 79% ^(^ ^21^ ^)^ to 83.3% ^(^ ^18^ ^)^ of the sampled tourniquets were often used by more than one health professional during peripheral venipuncture. Additionally, in one study ^(^ ^30^ ^)^ 62% of the health professionals used a tourniquet from another colleague in blood collection, with 96% stating they lost their own.

Interestingly, different authors ^(^ ^22^ ^)^ found that 54.7% of the participants didn’t use their tourniquet with patients with a known infection. Likewise, 3% of the health professionals in another study ^(^ ^30^ ^)^ commented that they specifically used a different tourniquet when the patient was known to have a communicable infectious disease. However, other authors identified opposing practices ^(^ ^31^ ^)^ , since health professionals didn’t change their tourniquets while caring for patients that were already signaled with Methicillin-resistant *Staphylococcus aureus* (MRSA).

As regards to tourniquet decontamination, in one study, a mere 35.5% of the health professionals disinfected their tourniquets ^(^ ^22^ ^)^ . With similar results, authors in another study ^(^ ^24^ ^)^ found that 34% of all tourniquets sampled had been cleaned or disinfected before, however no systematized protocol was found by the authors.

In another research effort ^(^ ^18^ ^)^ 16.7% of the health professionals disinfected their tourniquets before and after peripheral venipuncture. However, 25% did it once per shift, 16.7% only after peripheral venipuncture and 8.3% per certain unspecific periods. However, no tourniquet decontamination protocol was in place, and 70% ethyl alcohol was used by 66.7% of the health professionals ^(^ ^18^ ^)^ . As identified by other authors ^(^ ^21^ ^)^ , only 23% of the inquired health professionals cleaned their tourniquets. However, the same authors found no uniformity in the methods employed to clean the non-disposable tourniquets used (e.g., laundering at home versus rubbing with alcohol wipes).

In one study, tourniquet reprocessing protocols were heterogeneous, with most professionals using disinfection wipes after each use and daily machine washing at 60 degrees ^(^ ^32^ ^)^ . Furthermore, one author ^(^ ^20^ ^)^ identified that 77.6% of the participants stated to disinfect their tourniquets while 32.7% only cleaned them with water and soap. Regarding those who disinfect their tourniquets, only 14% clean them with water and soap before ^(^ ^20^ ^)^ . According to the same author, the most frequently used products were 70% ethyl alcohol (50%), followed by common hand disinfectant (16%), chlorhexidine at 1.5m/V (6%) and cetrimide at 15% m/V (2%).

Overall, significant microbiological contamination rates were found, ranging between 10 ^(^ ^24^ ^)^ and 100% ^(^ ^21^ ^,^ ^25^ ^-^ ^26^ ^,^ ^30^ ^)^ of all collected tourniquets. Overall, *Staphylococcus* spp. was the most prevalent identified bacterial genus ^(^ ^18^ ^-^ ^22^ ^,^ ^24^ ^-^ ^32^ ^)^ , with *S. aureus* being the most frequently found bacterial specie ^(^ ^18^ ^-^ ^22^ ^,^ ^24^ ^-^ ^31^ ^)^ . With equal clinical relevance, tourniquets were also shown to be contaminated with *Enterococcus* spp. ^(^ ^20^ ^,^ ^26^ ^)^ and Gram negative bacilli such as *Klebsiella* , *Pseudomonas* , *Escherichia coli* and *Acinetobacter baumannii* ^(^ ^28^ ^-^ ^29^ ^,^ ^31^ ^)^ . Antimicrobial resistance to methicillin emerged as the most frequently identified across all included studies ^(^ ^19^ ^-^ ^22^ ^,^ ^24^ ^,^ ^27^ ^,^ ^28^ ^,^ ^31^ ^)^ with rates varying between 2.2 ^(^ ^27^ ^)^ and 44.1% ^(^ ^31^ ^)^

Six out of the fifteen included studies analyzed other professional practices that could influence the use of a tourniquet during peripheral venipuncture, highlighting hand hygiene and the use of gloves as two practices that, when not followed in accordance with the international recommendations, contribute to the microbiological contamination of these medical devices.

Regarding hand hygiene during peripheral venipuncture and inherent tourniquet use, in one study, 88% of the health professionals stated they didn’t wash their hands between patients ^(^ ^29^ ^)^ . Similarly, in another study ^(^ ^27^ ^)^ , health professionals only washed their hands after glove removal.

Three studies focused on hand washing before and after peripheral venipuncture ^(^ ^26^ ^,^ ^30^ ^-^ ^31^ ^)^ . In one study ^(^ ^26^ ^)^ , 19.4% of the health professionals didn’t attempt to perform hand hygiene (with water and soap or alcohol gel) before performing peripheral venipuncture, while 43.5% did it occasionally and only 37.1% performed it every time. However, after performing peripheral venipuncture, 61.3% washed their hands consistently, 37.1% washed them occasionally and 1.6% never washed them ^(^ ^26^ ^)^ .

Similarly, in another research effort ^(^ ^30^ ^)^ only 42% of the participants in their study washed their hands before performing venipuncture (72% of these consistently), while 45% washed their hands after the procedure (53% of these consistently). Moreover, in an alternative study ^(^ ^31^ ^)^ , the authors initially questioned the participants about their adherence to hand hygiene before and after peripheral venipuncture, and later observed their practices. Regarding the participants’ answers, 75.3% stated that they performed hand hygiene before and after venipuncture, with only 26.9% doing it consistently ^(^ ^31^ ^)^ . However, 14% stated they only perform hand hygiene after peripheral venipuncture. In the observational period, the authors verified that 45.1% of the nurses performed hand hygiene before and 23.1% after the procedure ^(^ ^31^ ^)^ .

The use of gloves during peripheral venipuncture was analyzed in six studies ^(^ ^23^ ^,^ ^26^ ^-^ ^27^ ^,^ ^29^ ^-^ ^31^ ^)^ . In one study ^(^ ^23^ ^)^ , only 37% (42/114) of the health professionals routinely wore gloves in tourniquet use. With different results, in an additional study, gloves were only used to handle blood samples from patients in isolation ^(^ ^27^ ^)^ . Other authors found that the 48% of the included participants always wore gloves, while 27% used them occasionally ^(^ ^30^ ^)^ . Similar results were found in another work ^(^ ^26^ ^)^ , with 67.7% of the health professionals never wearing gloves while performing peripheral venipuncture, 27.4% wear them occasionally and 4.9% wear them consistently.

In another research effort ^(^ ^31^ ^)^ , 35.5% of the included health professionals reported using gloves during peripheral venipuncture, while 28% didn’t. In the same study, 51.6% of the professionals reported changing gloves between different patients ^(^ ^31^ ^)^ . However, during the observational period, 58.2% of the professionals didn’t use gloves, and 21.4% didn’t changed them between patients or between different procedures with the same patient ^(^ ^31^ ^)^ . Additionally, another authors ^(^ ^29^ ^)^ found that 92% of the included participants in their study didn’t discard their gloves between different patients.

## Discussion

The purpose of this scoping review was to map the evidence from studies that focused on health professionals’ practices related with tourniquet use during peripheral venipuncture. To meet this goal, 15 primary studies were included in this review. Although the inclusion of studies in this review did not limit the year or setting of publication, the included studies were published after 1986, in diverse international settings, which indicates that the scientific and professional community recognize the need to analyze such practices given inherent risks to quality and safety.

Transversally, the focus on the practices of the professionals in tourniquet use was not consensual among authors. We verified that some studies focused on tourniquet characteristics ^(^ ^18^ ^-^ ^21^ ^,^ ^23^ ^,^ ^25^ ^,^ ^29^ ^)^ , reuse of this device between patients ^(^ ^19^ ^,^ ^21^ ^-^ ^22^ ^,^ ^29^ ^)^ , decontamination practices ^(^ ^18^ ^,^ ^20^ ^-^ ^22^ ^,^ ^24^ ^,^ ^32^ ^)^ and tourniquet sharing between professionals ^(^ ^18^ ^,^ ^20^ ^-^ ^21^ ^,^ ^30^ ^)^ , while other studies also analyzed associated professional practices that could interfere with tourniquet use, such as hand hygiene ^(^ ^26^ ^-^ ^27^ ^,^ ^29^ ^-^ ^31^ ^)^ and the use of gloves ^(^ ^23^ ^,^ ^26^ ^-^ ^27^ ^,^ ^29^ ^-^ ^31^ ^)^ during peripheral venipuncture. Therefore, mapping the existing evidence on this subject was purely described, and no further comments can be made regarding divergent practices between different professional categories or geographical and clinical settings.

In this review, some studies ^(^ ^18^ ^-^ ^21^ ^,^ ^23^ ^,^ ^25^ ^,^ ^29^ ^)^ have indicated that reusable tourniquets are employed during peripheral venipuncture (made of common plastic, silicone, velcro or fabric), although the use of other medical devices (e.g., gloves ^(^ ^18^ ^)^ , gauze ^(^ ^25^ ^)^ and drains ^(^ ^19^ ^)^ ) or techniques (e.g., manual pressure ^(^ ^25^ ^)^ ) were also reported as an alternative practice by health professionals. This reality hampers any feasible attempts of an aseptic technique during peripheral venipuncture, constituting a risk to the quality and safety of care provided to patients ^(^ ^5^ ^)^ .

According to the Spaulding classification ^(^ ^12^ ^)^ , tourniquets can be classified as non-critical medical devices since they are used by health professionals in areas with intact skin. However, given the proximity between the area of tourniquet application and the puncture site (an entry point into the patient’s bloodstream), and the professional’s need to manipulate it throughout the procedure (e.g., releasing the tourniquet after locating a vein), the risk of microbial migration is increased. Therefore, we consider that tourniquets should be considered and used in clinical practice as semi-critical devices, requiring high level reprocessing practices ^(^ ^12^ ^)^ .

In this review, all the included studies ^(^ ^18^ ^-^ ^32^ ^)^ evidenced considerable microbiological contamination rates and bacterial diversity, which may be explained by the overall lack of appropriated reprocessing practices before and after tourniquet use during peripheral venipuncture. International recommendations state that health organizations should ensure that the reusable tourniquets can be decontaminated as per manufacturers guidelines between patient use ^(^ ^11^ ^,^ ^13^ ^)^ . Nevertheless, in none of the included studies clear institutional tourniquet decontamination protocols have been reported. This may explain the existence of different practices, not only in terms of their systematization (frequency and duration), but also regarding the technique and cleaning/disinfection agents used. Nonetheless, the absence of these data makes it impossible to truly discuss whether health professionals adopt these practices despite the existence of evidence and defined protocols for this purpose in their units/departments.

Such professional practices may constitute a potential risk for microbial cross-contamination between patients due to inefficient tourniquet decontamination, since factors like material contamination level, concentration and exposure time of the applied disinfectant, physical characteristics of the clinical material (cracks, lumens, etc.), presence of biofilm, temperature and solution pH level may also affect the effectiveness of the decontamination process ^(^ ^33^ ^)^ .

Only one study evidenced that a small number of health professionals attempted to clean their tourniquets with water and soap before disinfecting them ^(^ ^20^ ^)^ . Moreover, in this review, we found that a significant number of health professionals disinfected their tourniquets with alcohol-based products ^(^ ^18^ ^,^ ^20^ ^,^ ^32^ ^)^ , which do not penetrate well into protein-based matter ^(^ ^12^ ^)^ . These results are noteworthy, since the chemical action of commonly used agents for the disinfection of medical devices is counteracted by the presence of organic matter like blood ^(^ ^14^ ^)^ .

Moreover, associated professional practices such as lack of systematized hand hygiene and underuse of gloves during peripheral venipuncture may have contributed to the microbiological contamination of these devices, or vice-versa. These results pose significant risk to patient safety and care, especially when considering that the tourniquets included in this review were contaminated with pathogenic species such as *S.* aureus ^(^ ^18^ ^-^ ^22^ ^,^ ^24^ ^-^ ^31^ ^)^ *, Klebsiella, Pseudomonas, Escherichia coli* and *Acinetobacter baumanni* ^(^ ^28^ ^-^ ^29^ ^,^ ^31^ ^)^ , with significant antimicrobial resistances, which can negatively impact patient clinical outcomes ^(^ ^19^ ^-^ ^22^ ^,^ ^24^ ^,^ ^27^ ^-^ ^28^ ^,^ ^31^ ^)^ . Such risks are aggravated when we consider that a significant number of the tourniquets analyzed in this review were used in clinical settings such as intensive care units ^(^ ^18^ ^,^ ^23^ ^,^ ^27^ ^,^ ^31^ ^)^ , operating theatres ^(^ ^18^ ^,^ ^26^ ^-^ ^27^ ^)^ , dialysis units ^(^ ^23^ ^,^ ^28^ ^)^ , maternal and pediatric units ^(^ ^18^ ^,^ ^25^ ^)^ , where we commonly find patients whose already weakened clinical condition predisposes them to nosocomial infections.

Nevertheless, international recommendations clearly state that tourniquets made of materials which cannot be properly decontaminated should not be used and disposable equipment should be implemented wherever possible ^(^ ^11^ ^,^ ^13^ ^)^ . In this review, prior to any intervention by the authors, no study was found where single-use tourniquets were available in their clinical settings. However, later on in one study, single-use disposable tourniquets were introduced to health professionals, but only 27% started and continued to use them ^(^ ^25^ ^)^ . Therefore, even if the re-use of tourniquets is justified by some participants because of insufficient resources in their units ^(^ ^29^ ^)^ , the introduction of single-use disposable devices may not be enough to make professionals aware of the risk associated with tourniquet use during peripheral venipuncture.

This result may explain why changing current professional practices related to tourniquet use during peripheral venipuncture is not a linear intervention, and should attend to more variables at an individual (e.g., perception of risk, motivation and workload and ratios) and organizational level (e.g., costs of acquisition, supply periods and organizational structural barriers).

As an example, some authors ^(^ ^23^ ^,^ ^26^ ^-^ ^27^ ^,^ ^29^ ^-^ ^31^ ^)^ identified two main professional practices during peripheral venipuncture that can contribute to tourniquet contamination: hand hygiene and glove use. International guidelines recommend that hand hygiene should be performed before inserting a peripheral vascular catheter (or even, before contacting a patient), after contact with the patient’s intact or nonintact skin (or with body fluids or excretions, such as blood) and after contact with inanimate objects (including medical equipment such as tourniquets) in the immediate vicinity of the patient ^(^ ^4^ ^-^ ^5^ ^,^ ^13^ ^)^ . However, reported practices evidence that hand hygiene moments aren’t strictly followed by health professionals. Before peripheral venipuncture, observed adherence rates varied from 37.1% ^(^ ^26^ ^)^ to 45.1% ^(^ ^31^ ^)^ , which further increases the chances of tourniquet contamination even before being in contact with the patient’s skin, posing a risk of cross-contamination. On the same line of thought, after peripheral venipuncture, observed adherence rates to hand hygiene varied between 23.1% ^(^ ^31^ ^)^ and 61.3% ^(^ ^26^ ^)^ . It is worth mentioning that higher rates of non-conformity were found in other studies ^(^ ^27^ ^,^ ^29^ ^)^ , but these were self-reported by health professionals, and might not correspond to their actual practice in clinical setting.

Using appropriate personal protective equipment, such as well-fitting gloves, during peripheral venipuncture is a well-established international recommendation. However, most studies verified that gloves weren’t changed in patient care when torn or heavily contaminated, or if moving from a contaminated body site to a clean body site ^(^ ^26^ ^,^ ^29^ ^,^ ^31^ ^)^ .

Additionally, some authors verified that the same pair of gloves was used when caring for more than one patient ^(^ ^29^ ^,^ ^31^ ^)^ , hindering care safety and quality due to the high risk of cross-contamination ^(^ ^5^ ^)^ . Glove use should be timely and applied immediately before and removed immediately after procedures involving peripheral venipuncture, taking in consideration not only the procedure being undertaken, but also the contact with susceptible sites or devices such as tourniquets ^(^ ^4^ ^,^ ^13^ ^)^ .

Therefore, the outlined evidence resulting from this scoping review proves that it is essential to have a quality assurance/management system in place regarding the use and reprocessing of medical devices that can pose an health risk to patients and health professionals ^(^ ^12^ ^)^ . Clinical settings must have record-keeping policies in place, which require professionals to document the type of tourniquet used during venipuncture and what precautions have been taken to prevent cross-contamination. Clinical settings must have a risk management and audit system in place ^(^ ^12^ ^)^ , ensuring that non-conformances, incidents and errors related to tourniquet use during peripheral venipuncture are identified promptly, investigated, evaluated and documented.

Furthermore, tourniquet contamination may pose an health risk to the professionals themselves ^(^ ^20^ ^)^ , given the direct and recurrent contact between the device and their hands. Tourniquets are often kept in the professional’s uniform pockets ^(^ ^20^ ^)^ or in a place of free and shared access between professionals, such as intravenous carts or cabinet drawers ^(^ ^20^ ^-^ ^21^ ^,^ ^23^ ^,^ ^26^ ^,^ ^30^ ^-^ ^32^ ^)^ , which may constitute an occupational health risk. Thus, specific safety policies and procedures should be implemented, focusing on staff education and training regarding the risks associated with the use of this device, with the breakdown of the current realities in the clinical settings that sustain the results found. Nevertheless, it is also important to emphasize the central role of educational institutions in the education of nursing and medical students in the use of medical devices that can pose a risk to patient’s clinical condition, such as the tourniquet during peripheral venipuncture, since underwhelming results were found in studies that included a large student sample ^(^ ^29^ ^-^ ^30^ ^)^ .

This scoping review presents as limitations the inclusion of studies in only four languages (English, Portuguese, French and Spanish) and the inclusion of studies from the selected six databases and three repositories, which may have limited the access to other relevant data.

We hope that from the outlined realities identified throughout this review new lines of research may emerge, with contributions beyond the thematic awareness, but as a support for the reformulation and restructuring of existing practices, devices and policies in the clinical settings related to tourniquet use during the peripheral venipuncture. For that reason, it would be important to produce scientific evidence on the impact of certain observed practices (e.g., recurrent tourniquet disinfection between users) in the potential contamination of these devices and associated complications verified in procedures with peripheral venipuncture. In addition, further research should be done in order to correlate the microbiological contamination of the tourniquets use during peripheral venipuncture and the patient’s puncture site (and catheter tip, when reporting to peripheral catheterization), establishing if the genetic profile of the found bacteria is identical.

## Conclusion

This scoping review allowed the mapping of professional practices related to tourniquet use during peripheral venipuncture. The gathered evidence suggests that health professionals are not uniform in their approach and do not follow the principles established in international guidelines, thus contributing to tourniquet contamination, with potential implications on the quality, safety and effectiveness of the care provided to the patient.

It should be highlighted that tourniquet contamination does not appear to be a concern of professionals during peripheral venipuncture, one of the most frequent procedures in clinical practice, since only a limited number of studies identified practices related to tourniquet disinfection, substitution after single-use or risk-related measures performed in specific cases (e.g., patients in isolation).

Therefore, given the underwhelming results found in the literature, we hope that the mapping of current practice motivates new research efforts that aim to analyze the impact of implementing single-use tourniquets (e.g., cost-effect) or professional education and training regarding tourniquet reprocessing practices (e.g., disinfection and single-patient allocation) on the contamination of the puncture site and peripheral venous catheter.
